# Body Mass Index, Weight Loss, and Mortality Risk in Advanced-Stage Non-Small Cell Lung Cancer Patients: A Focus on EGFR Mutation

**DOI:** 10.3390/nu13113761

**Published:** 2021-10-24

**Authors:** Yu-Mu Chen, Chien-Hao Lai, Chiung-Yu Lin, Yi-Hsuan Tsai, Ya-Chun Chang, Hung-Chen Chen, Chia-Cheng Tseng, Huang-Chih Chang, Kuo-Tung Huang, Yung-Che Chen, Wen-Feng Fang, Chin-Chou Wang, Tung-Ying Chao, Meng-Chih Lin

**Affiliations:** 1 Department of Internal Medicine, Division of Pulmonary and Critical Care Medicine, College of Medicine, Chang Gung University, Kaohsiung Chang Gung Memorial Hospital, Niaosung, Kaohsiung 83301, Taiwan; blackie@cgmh.org.tw (Y.-M.C.); lieo@cgmh.org.tw (C.-H.L.); chiungyu@cgmh.org.tw (C.-Y.L.); yhtsai@cgmh.org.tw (Y.-H.T.); y7817@cgmh.org.tw (Y.-C.C.); chc1106@cgmh.org.tw (H.-C.C.); cctseng@cgmh.org.tw (C.-C.T.); kuan2101@yahoo.com.tw (H.-C.C.); jelly@cgmh.org.tw (K.-T.H.); yungchechen@yahoo.com.tw (Y.-C.C.); wenfeng@cgmh.org.tw (W.-F.F.); ccwang52@cgmh.org.tw (C.-C.W.); tychao@cgmh.org.tw (T.-Y.C.); 2 Graduate Institute of Clinical Medical Sciences, College of Medicine, Chang Gung University, Kaohsiung Chang Gung Memorial Hospital, Kaohsiung 83301, Taiwan; 3 Department of Respiratory Therapy, College of Medicine, Chang Gung University, Kaohsiung Chang Gung Memorial Hospital, Kaohsiung 83301, Taiwan; 4 Department of Respiratory Care, Chang Gung University of Science and Technology, Chiayi 61363, Taiwan; 5 Respiratory Center of Excellence, Chang Gung Medical Insititute, Kaohsiung 83301, Taiwan

**Keywords:** body mass index, weight loss, non-small cell lung cancer, tyrosine kinase inhibitor

## Abstract

Body mass index (BMI) influences the prognosis of patients with non-small cell lung cancer (NSCLC), including both early-stage and late-stage NSCLC patients that are undergoing chemotherapies. However, earlier research on the relationship between BMI and survival in patients taking epidermal growth factor receptor (EGFR)-tyrosine kinase inhibitors (TKIs) yielded contradictory results. These publications either had a limited number of patients or were getting TKIs in various lines of therapy, which might explain why the outcomes were contradictory. As a result, we undertook retrospective study to examine the effect of BMI on survival outcomes in patients with advanced EGFR mutant NSCLC receiving first-line EGFR-TKIs. We also compared the findings to those with wild-type EGFR. Between November 2010 and March 2014, 513 patients with advanced NSCLC were enrolled in the study. According to the adjusted BMI cut-off point for Asia, 35 out of 513 (6.8%) patients were underweight (BMI < 18.5 kg/m^2^), whereas 197 (38.4%) were overweight (BMI > 24 kg/m^2^). Overweight patients with wild-type EGFR exhibited longer progression-free survival (4.6 vs. 2.1 months, *p* = 0.003) and overall survival (OS) (8.9 vs. 4.3 months, *p* = 0.003) than underweight patients. Overweight patients with EGFR mutations had a longer OS than normal-weight patients (23.0 vs. 20.2 months, *p* = 0.025). Bodyweight reduction was related to a shorter OS in both the mutant EGFR patients (17.1 vs. 30.5 months, *p* < 0.001) and the wild-type EGFR patients (7.8 vs. 18.7 months, *p* < 0.001). In conclusion, advanced stages NSCLC patients with a lower BMI and early weight loss had a worse outcome that was independent of EGFR mutation status.

## 1. Introduction

Lung cancer is the most frequent disease that is diagnosed and is the main cause of cancer mortality globally [1]. Being overweight has been identified as a risk factor for the majority of malignancies [2,3,4,5]. The incidence of lung cancer, on the other hand, was found to have an inverse relationship with BMI [2,3,4,5]. Higher body mass index (BMI) has been linked to a better prognosis in patients with early-stage non-small cell lung cancer (NSCLC) [6], as well as in NSCLC patients who were undergoing chemotherapies [7,8,9]. Lung cancer treatments have advanced considerably in recent years [10]. The discovery of EGFR-tyrosine kinase inhibitors (TKIs) enhanced the life quality and survival in advanced NSCLC patients with sensitive EGFR mutations [11,12,13]. Previous research has discovered several predictors of EGFR-TKI treatment response, including performance status and metastatic locations [14]. Previous research, however, found conflicting results regarding the baseline BMI and the survival of patients taking EGFR-TKIs. One study found that a lower BMI was linked to a longer progression-free survival (PFS) in EGFR-TKI patients [15], however, another study found that a lower BMI was associated with worse survival results [16]. The study found that BMI did not affect the effectiveness of EGFR-TKIs [17]. These trials either were comprised of a limited number of patients or those who got TKIs after first-line treatment. As a result, we conducted a retrospective study to assess the influence of BMI and bodyweight reduction on survival outcomes in patients with EGFR mutant, advanced-stage NSCLC who had received first-line EGFR-TKIs. We also compared the findings to patients with wild-type EGFR.

## 2. Materials and Methods

### 2.1. Study Population

A retrospective study was performed at Kaohsiung Chang Gung Memorial Hospital, a university-affiliated medical facility, between 1 November 2010, and 30 March 2014. Patients were followed up on until 31 December 2015. Patients that were over the age of 18 with cytologically or histologically confirmed, newly diagnosed stage IIIB or IV NSCLC, and who had EGFR mutation testing were eligible. Patients who did not have baseline BMI data or were lost to follow-up were excluded. Within one month after starting therapy, baseline clinical profiles, chest computed tomography, bone scintigraphy, and brain magnetic resonance imaging were all evaluated. We defined underweight and overweight patients using a modified BMI cut-off criteria for Asians [18,19,20]. According to BMI, patients were classified into three groups: underweight (BMI 18.5 < kg/m^2^), normal-weight (BMI 18.5–24 kg/m^2^), and overweight (BMI > 24 kg/m^2^). A bodyweight loss of more than 5% was classified when it occurred within three months of the diagnosis of lung cancer [21]. The study was authorized by the Kaohsiung Chang Gung Memorial Hospital’s Institutional Review Board, with permission number 104–7011C. Informed consent was not required.

### 2.2. Testing of EGFR Mutation Status

Bronchoscopy, pleural effusion cytology, computed tomography (CT)-guided biopsy, and surgical techniques were used to acquire the tumor tissue. The EGFR mutation was frequently tested in patients with a variety of histologic subtypes, including adenocarcinoma, adenosquamous cell carcinoma, large cell carcinoma, and NSCLC that was not otherwise specified. It was also performed on patients with squamous cell carcinoma who had never smoked. Testing was evaluated with the QIAGEN EGFR RGQ PCR Kit’s SCORPIONS and Amplified Refractory Mutation System (ARMS) [22]. The EGFR testing results were obtained from the patient’s health records.

### 2.3. Evaluation of Disease Status

Every 2–4 weeks, chest radiography was performed to assess the tumor response, and every 2–3 months, a chest CT was employed. The disease state was assessed by the physician using the Response evaluation criteria in solid tumors criteria 1.1 [23].

The primary and secondary endpoints were PFS and overall survival (OS). PFS was defined as the time between the first day of EGFR-TKI or chemotherapy treatment and disease progression, the last day of follow-up, or death before tumor progression. The time from the first day of EGFR-TKI or chemotherapy and death or the last follow-up was referred to as the OS.

### 2.4. Statistical Analyses

MedCalc was used for statistical analysis (Version 14). A Student’s *t*-test or Mann–Whitney U test was used to evaluate the continuous variables while a Chi-square test or Fisher’s exact test was used to evaluate the categorical variables between patients with or without EGFR mutation. A one-way ANOVA was used to evaluate the continuous variables in the three BMI subgroups. A Chi-square test or Fisher’s exact test was used to evaluate categorical variables in the three BMI subgroups. PFS was the date of the initiation of first-line treatment to disease progression or death, censoring at the date of the last follow-up without disease progression. OS was the date of diagnosis to that of death, censoring at the date of the last follow-up in alive patients. The Kaplan–Meier estimate and the log-rank test were used to evaluate the PFS and OS. A *p* value of < 0.05 was considered statistically significant. 

## 3. Results

### 3.1. Patient Characteristics

**Figure 1:**
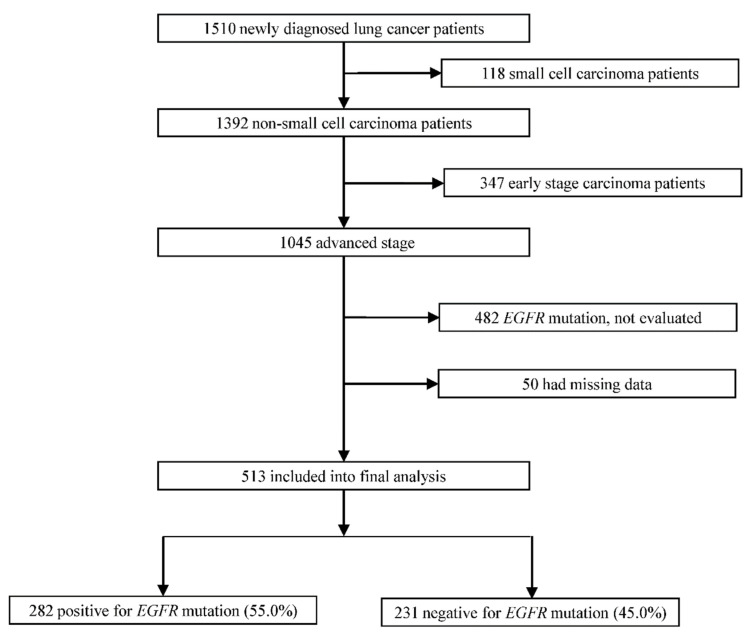
The flow diagram depicting the patient’s enrollment scheme.

A total of 513 advanced NSCLC patients who were tested for EGFR mutation status were included in the final study out of 1510 lung cancer patients (Figure 1). The EGFR mutation was found in 54.9% (*n* = 282) of the patients. All NSCLC patients with EGFR mutations received first-line EGFR-TKIs, while 211 out of 231 patients without EGFR mutations received chemotherapy. The median duration between follow-ups was 33.0 months, while the longest time between follow-ups was 54.8 months. After the follow-up period, 22.8% of the patients (117/513) were still alive. PFS and OS were 7.0 and 14.4 months, respectively, in all patients, 3.6 and 8.4 months in patients with wild-type EGFR, respectively, and 10.9 and 21.0 months in patients with EGFR mutation, respectively. Table 1 displays the baseline clinical characteristics of all of the patients. 

### 3.2. Clinical Characteristics between NSCLC Patients with Wild Type and Mutant EGFR

When comparing the baseline clinical characteristics in patients with the wild type and mutant EGFR, there were significant differences in BMI (patients with wild type vs. mutant EGFR: 22.6 ± 3.6 vs. 23.3 ± 3.7, *p* = 0.022), sex (wild type vs. mutant EGFR: 41.6% women vs. 57.1%, *p* < 0.001), DM (wild type vs. mutant EGFR: 18.6 vs. 4.3%, *p* < 0.001), COPD (wild type vs. mutant EGFR: 11.7 vs. 5.0%, *p* = 0.005), smoking history (wild type vs. mutant EGFR: 45.9% vs. 31.2%, *p* = 0.001), tumors of adenocarcinoma histology (wild type vs. mutant EGFR: 76.2% vs. 91.8%, *p* < 0.001), and the number of distal metastasis (wild type vs. mutant EGFR: 1.6 ± 1.1 vs. 1.4 ± 1.0, *p* = 0.041) (Table 1). There were no significant differences in the age, ECOG PS, tumor stage, or sites of distant metastases including brain, bone, liver, and pleura between patients with the wild type and mutant EGFR (Table 1).

### 3.3. Clinical Characteristics of Underweight, Normal-Weight, and Overweight Patients 

Underweight NSCLC patients were more likely to have COPD (*p* = 0.019) (Table 2). In the subgroup analyses, underweight lung cancer patients were older in those with wild type EGFR (Table 2). More COPD, poor ECOG PS, and more liver metastases in underweight lung cancer were only noted in EGFR mutant patients (Table 2). Normal-weight patients were more likely to have never been a smoker in EGFR mutant patients, but not in EGFR wild-type patients. (Table 2) Other baseline parameters did not differ statistically between underweight, normal-weight, and overweight patients (Table 2). 

### 3.4. Influence of Baseline BMI on Outcomes of NSCLC Patients with or without EGFR Mutation

**Figure 2:**
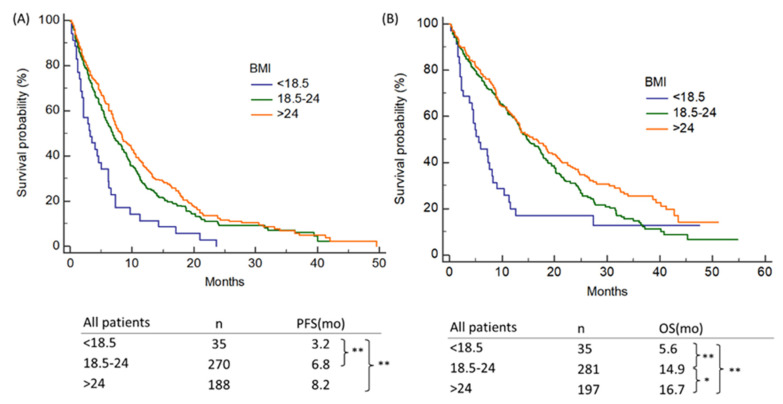
Influence of the baseline BMI on (**A**) PFS and (**B**) OS of NSCLC patients. BMI—body mass index; PFS—progression-free survival; OS—overall survival; NSCLC—non-small cell lung cancer; Note: * *p* < 0.05, ** *p* < 0.001.

Overweight patients exhibited a longer PFS than the underweight patients (8.2 vs. 3.2 months, *p* < 0.001) across all advanced NSCLC patients. (See Figure 2A.) There was no statistical significant difference in PFS between the patients who were overweight and those who were normal-weight. Overweight people lived longer than the normal-weight and underweight people. (16.7 months vs. 14.9 months, *p* = 0.047; and 14.9 months vs. 5.6 months, *p* 0.001, respectively) (Figure 2B). Patients who were a normal weight had a longer PFS (6.8 vs. 3.2 months, *p* < 0.001) and OS (14.9 months vs. 5.6 months, *p* = 0.007) than those who were underweight. 

### 3.5. Influence of Baseline BMI on Outcomes of NSCLC Patients with Wild-Type EGFR Status

**Figure 3:**
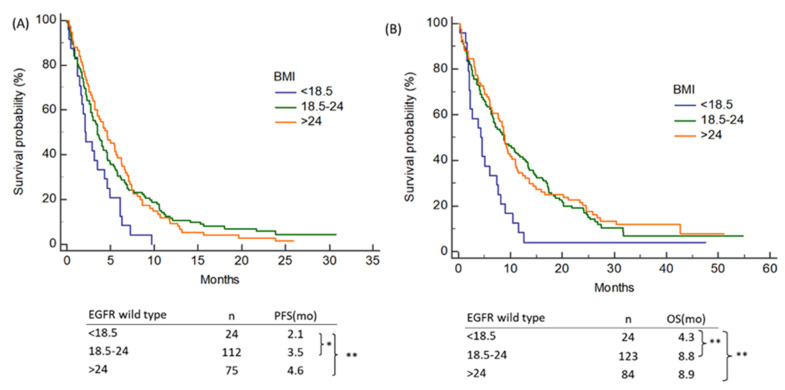
Influence of the baseline BMI on (**A**) PFS and (**B**) OS of NSCLC patients with wild-type EGFR status. BMI—body mass index; PFS—progression-free survival; OS—overall survival; NSCLC—non-small cell lung cancer; EGFR—epidermal growth factor receptor; Note: * *p* < 0.05, ** *p* < 0.001.

Overweight NSCLC patients with the wild-type EGFR status exhibited longer PFS and OS than the underweight patients (4.6 vs. 2.1 months, *p* = 0.003; 8.9 vs. 4.3 months, p = 0.003, respectively) (Figure 3A,B) Patients who were a normal weight had a longer PFS and OS than those who were underweight (3.5 vs. 2.1 months, *p* = 0.018; 8.8 vs. 4.3 months, *p* = 0.003, respectively) (Figure 3A). There was no statistically significant difference in the PFS or OS between patients who were overweight or normal-weight. 

### 3.6. Influence of Baseline BMI on Outcomes of NSCLC Patients with EGFR Mutation

**Figure 4:**
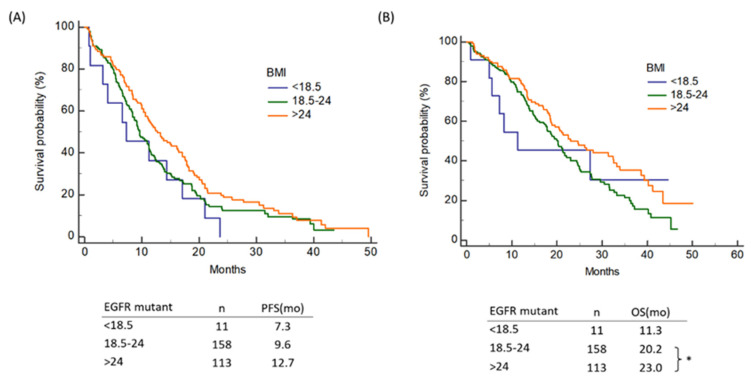
Influence of the baseline BMI on (**A**) PFS and (**B**) OS of NSCLC patients with EGFR mutation. BMI—body mass index; PFS—progression-free survival; OS—overall survival; NSCLC—non-small cell lung cancer; EGFR—epidermal growth factor receptor; Note: * *p* < 0.05.

There was no difference in PFS between overweight, normal-weight, and underweight patients among the EGFR mutant patients (Figure 4A). The overweight patients with EGFR mutations, on the other hand, had a higher life expectancy than the normal-weight patients. (*p* = 0.025, 23.0 vs. 20.2 months) (See Figure 4B.) 

### 3.7. Influence of Weight Loss on Outcomes of NSCLC Patients

**Figure 5:**
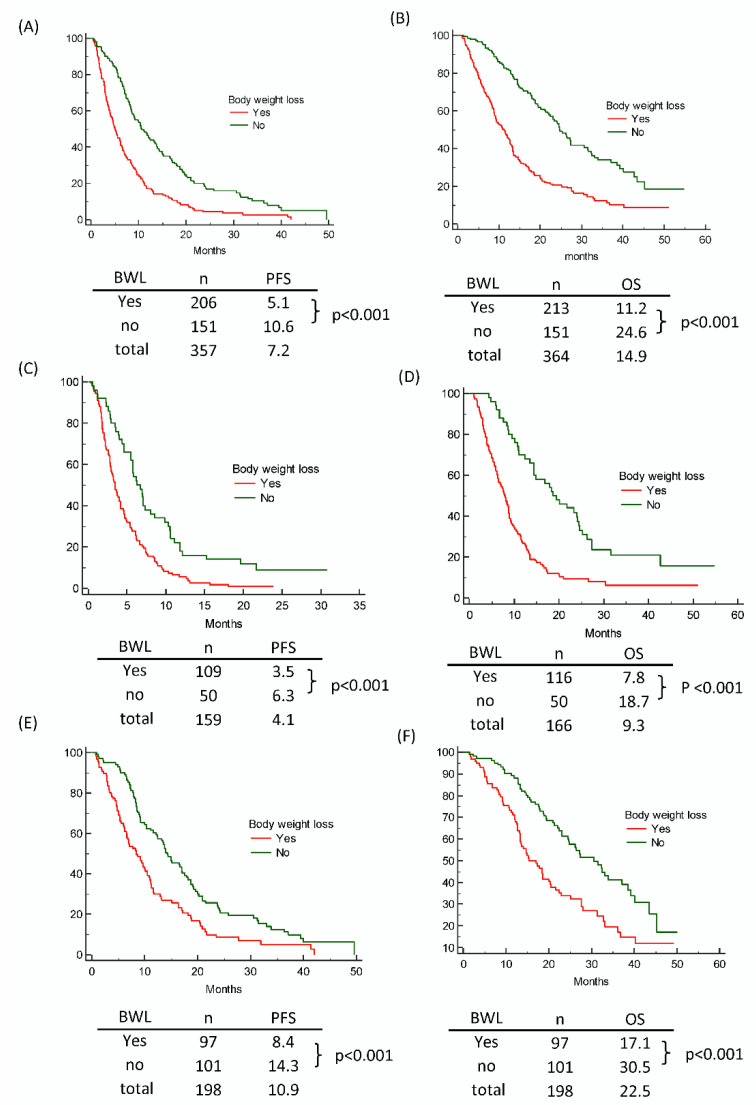
Influence of the body weight loss on (**A**) PFS and (**B**) OS of all NSCLC patients; (**C**) PFS, and (**D**) OS of NSCLC patients with wild-type EGFR status; (**E**) PFS and (**F**) OS of NSCLC patients with EGFR mutation. BMI—body mass index; PFS—progression-free survival; OS—overall survival; NSCLC—non-small cell lung cancer; EGFR—epidermal growth factor receptor.

Among the 513 patients with available baseline BMI data, 364 had follow-up BMI data within 3 months of a lung cancer diagnosis. A total of 264 (58.5%) of the 364 patients lost weight. NSCLC patients with wild-type EGFR were more likely to lose bodyweight than NSCLC patients with mutant EGFR (116/166 [69.9%] vs. 97/198 [49.0%], respectively, *p* < 0.0001). A reduction in bodyweight was linked with shorter OS in both patients with mutant EGFR (17.1 vs. 30.5 months, *p* < 0.001) and patients with wild-type EGFR (7.8 vs. 18.7 months, *p* < 0.001). (Figure 5)

## 4. Discussion

Our research found that patients with EGFR mutations who were overweight had a higher chance of survival. Previous research on the relationship between baseline BMI and survival in patients that were receiving EGFR-TKIs yielded contradictory results. A lower BMI was related to a worse survival result in one study [16], which was consistent with our findings. Another study with a small number of patients found that having a higher BMI was linked with shorter PFS [15]. The author hypothesized that the outcome was due to greater EGFR-TKI blood concentrations in the smaller body size group. In a randomized dosage escalation study with gefitinib, however, the higher dose group had higher mean plasma concentrations but the same response rate and survival outcomes [24].

A higher BMI was observed to be related to a better prognosis in NSCLC patients who were undergoing chemotherapies, which was consistent with prior research. [7,8,9]. Surprisingly, one of these studies stated that the protective benefit of a high BMI is only temporary [7]. An obese patients’ risk of all-cause death increased substantially once they lived for more than 16 months [7]. Our research also found that overweight patients who lived for more than 18 months had a higher death rate. Several ideas have been advanced, including the possibility of a synergy between the peroxisome proliferator-activated receptor (PPAR) ligands and platinum-based medicines in first-line treatment [25]. Rapid progression following the absence of platinum-based agents in subsequent lines of therapy were due to a decrease in the synergy between platinum-based agents and PPAR ligands. Another theory was that overweight individuals were more likely to be given anti-diabetic medications [26]. Interactions with anti-diabetic medications may have contributed to their improved prognosis in their early cancer treatment course [27].

Our research found that a bodyweight reduction was linked with shorter OS in both patients with mutant EGFR (17.1 vs. 30.5 months, *p* < 0.001) and those with wild-type EGFR (7.8 vs. 18.7 months, *p* < 0.001). When comparing the NSCLC patients with the wild-type EGFR to those with mutant EGFR, our study found that individuals with the wild-type EGFR were more likely to experience early bodyweight loss. Previous research has indicated that cancer patients who lose weight have a worse prognosis than those who do not [28,29,30]. Pre-diagnosed weight reduction was found to be a better predictor of survival in lung cancer patients [31]. In recent research [32], weight loss during definitive radiotherapy was observed to result in a worse OS rate in patients with locally advanced NSCLC. Previous research found that a weight reduction at presentation had a negative influence on the survival outcomes in patients with EGFR-mutant advanced NSCLC that was treated with first-line EGFR-TKI [33]. Weight loss, independent of EGFR mutation status, was linked with a poor outcome in our research. Several weight loss theories have been proposed, including but not limited to systemic inflammation and the microbiota-muscle axis [34,35,36]. Several treatments enhanced the quality of life in cancer patients with anorexia or cachexia [37,38,39]. In cancer cachexia, oral nutritional interventions such as megestrol acetate and anamorelin enhanced the quality of life [39]. Some treatments, such as humanized monoclonal antibodies targeting human IL-6, histone deacetylase inhibitors, and Toll-like receptor agonists, showed promise in preclinical and clinical trials [40,41,42,43]. There were also some complementary medicines that were used for anorexia and cachexia in cancer patients, such as herbal medicine and acupuncture [37,44,45]. However, none of the existing cancer anorexia and cachexia treatment methods enhanced survival. [35,38,43,46]. Novel approaches are being advocated to combat cancer-related weight loss and consequent cancer cachexia-related death.

There were some limitations to our investigation. This retrospective analysis did not include other nutritional evaluations or comprehensive information regarding nutritional intervention [47,48,49,50]. In a future study, we will focus on dietary intervention and weight loss prevention.

## 5. Conclusions

NSCLC patients at advanced stages with a lower BMI and early weight loss had a worse outcome independent of EGFR mutation status.

## Figures and Tables

**Table 1:** Clinical characteristics of study patients (*n* = 513).

	All Patients (*n* = 513)	EGFR Wild Type (*n* = 231)	EGFR Mutant (*n* = 282)	*p* Value
Age (mean ± SD)	63.7 ± 12.3	63.0 ± 12.2	64.2 ± 12.3	0.249
BMI (mean ± SD)	23.0 ± 3.6	22.6 ± 3.6	23.3 ± 3.7	0.022
Sex				<0.001
Male	256 (49.9)	135 (58.4)	121 (42.9)	
Female	257 (50.1)	96 (41.6)	161 (57.1)	
DM	55 (10.7)	43 (18.6)	12 (4.3)	<0.001
COPD	41 (8.0)	27 (11.7)	14 (5.0)	0.005
Smoking history				0.001
Never	319 (62.2)	125 (54.1)	194 (68.8)	
Former/current	194 (37.8)	106 (45.9)	88 (31.2)	
Performance status				0.499
ECOG 0–2	471 (91.8)	210 (90.9)	261 (92.6)	
ECOG 3–4	42 (8.2)	21 (9.1)	21 (7.4)	
Stage				0.584
IIB	60 (11.7)	29 (12.6)	31 (11.0)	
IV	453 (88.3)	202 (87.4)	251 (89.0)	
Tumor type				<0.001
Adenocarcinoma	435 (84.8)	176 (76.2)	259 (91.8)	
Non-adenocarcinoma	78 (15.2)	55 (23.8)	23 (8.2)	
Brain metastases	116 (22.6)	53 (22.9)	63 (22.3)	0.871
Bone metastases	226 (44.1)	107 (46.3)	119 (42.2)	0.349
Liver metastases	70 (13.6)	35 (15.2)	35 (12.4)	0.368
Pleura metastases	185 (36.1)	76 (32.9)	109 (38.7)	0.177
No. of distal metastasis	1.5 ± 1.1	1.6 ± 1.1	1.4 ± 1.0	0.041

Abbreviations: BMI, body mass index; COPD, chronic obstructive pulmonary disease; DM, diabetes mellitus; ECOG, eastern cooperative oncology group; EGFR, epidermal growth factor receptor.

**Table 2:** Clinical characteristics of underweight, normal weight, and overweight patients.

	All Patients (*n* = 513)					EGFR Wild Type (*n* = 231)				EGFR Mutant (*n* = 282)			
	All Patients (*n* = 513)	BMI < 18.5 (*n* = 35)	BMI 18.5–24 (*n* = 281)	BMI > 24 (*n* = 197)	*p* Value	BMI < 18.5 (*n* = 24)	BMI 18.5–24 (*n* = 123)	BMI > 24 (*n* = 84)	*p* Value	BMI < 18.5 (*n* = 11)	BMI 18.5–24 (*n* = 158)	BMI > 24 (*n* = 113)	*p* Value
Age (mean ± SD)	63.7 ± 12.3	68.3 ± 14.5	62.8 ± 12.6	64.1 ± 11.2	0.056	68.7 ± 15.1	61.1 ± 12.4	64.2 ± 10.3	0.009	67.4 ± 13.7	64.2 ± 12.6	64.1 ± 11.8	0.695
Sex					0.648				0.672				0.112
Male	256 (49.9)	18 (51.4)	135 (48.0)	103 (52.3)		14 (58.3)	75 (61.0)	46 (54.8)		4 (36.4)	60 (38.0)	57 (50.4)	
Female	257 (50.1)	17 (48.6)	146 (52.0)	94 (47.7)		10 (41.7)	48 (39.0)	38 (45.2)		7 (63.6)	98 (62.0)	56 (49.6)	
COPD	41 (8.0)	6 (17.1)	15 (5.3)	20 (10.2)	0.019	4 (16.7)	11 (8.9)	12 (14.3)	0.364	2 (18.2)	4 (2.5)	8 (7.1)	0.028
DM	55 (10.7)	6 (17.1)	22 (7.8)	27 (13.7)	0.055	6 (25.0)	16 (13.0)	21 (25.0)	0.065	0 (0)	6 (3.8)	6 (5.3)	0.645
Performance status					0.140				0.407				0.030
ECOG 0–2	471 (91.8)	30 (85.7)	255 (90.7)	186 (94.4)		22 (91.7)	109 (88.6)	79 (94.0)		8 (72.7)	146(92.4)	107 (94.7)	
ECOG 3–4	42 (8.2)	5 (14.3)	26 (9.3)	11 (5.6)		2 (8.3)	14 (11.4)	5 (6.0)		3 (27.3)	12(7.6)	6 (5.3)	
Smoking history					0.081				0.919				0.028
Never	319 (62.2)	20 (57.1)	187 (66.5)	112 (56.9)		13 (54.2)	68 (55.3)	44 (52.4)		7 (63.6)	119(75.3)	68 (60.2)	
Former/current	194 (37.8)	15 (42.9)	94 (33.5)	85 (43.1)		11 (45.8)	55 (44.7)	40 (47.6)		4 (36.4)	39(24.7)	45 (39.8)	
Stage					0.404				0.729				0.090
IIB	60 (11.7)	5 (14.3)	28 (10.0)	27 (13.7)		2 (8.3)	15 (12.2)	12 (14.3)		3 (27.3)	13(8.2)	15 (13.3)	
IV	453 (88.3)	30 (85.7)	253 (90.0)	170 (86.3)		22 (91.7)	108 (87.8)	72 (85.7)		8 (72.7)	145(91.8)	98 (86.7)	
Tumor type					0.552				0.304				0.
Adenocarcinoma	435 (84.8)	27 (77.1)	237 (84.3)	171 (86.8)		16 (66.7)	92 (74.8)	68 (81.0)		11 (100.0)	145(91.8)	103 (91.2)	
Non-adenocarcinoma	78 (15.2)	8 (22.9)	44 (15.7)	26 (13.2)		8 (33.3)	31 (25.2)	16 (19.0)		0 (0)	13(8.2)	10 (8.8)	
EGFR Mutation					0.071								
Yes	282 (55.0)	11 (31.4)	158 (56.2)	113 (57.4)									
No	231 (44.0)	24 (68.6)	123 (43.8)	84 (42.6)									
EGFR Mutation type													0.985
Common										10 (90.9)	141 (89.2)	101 (89.4)	
Uncommon										1 (9.1)	17 (10.8)	12 (10.6)	
Brain metastases	116 (22.6)	7 (35.0)	50 (28.6)	61 (20.3)	0.391	5 (20.8)	33 (26.8)	15 (17.9)	0.310	2 (18.2)	37 (23.4)	24 (21.2)	0.863
Bone metastases	226 (44.1)	13 (65.0)	91 (52.0)	117 (39.0)	0.174	13(54.2)	61 (49.6)	33 (39.3)	0.247	5 (45.5)	70 (44.3)	44 (38.9)	0.661
Liver metastases	70 (13.6)	4 (20.0)	29 (16.6)	36 (12.0)	0.525	4(16.7)	13 (10.6)	18 (21.4)	0.099	3 (27.3)	24 (15.2)	8 (7.1)	0.043
Pleura metastases	185 (36.1)	11 (55.0)	58 (33.1)	108 (36.0)	0.467	13(54.2)	34(27.6)	29 (34.5)	0.038	3 (27.3)	65 (41.1)	41 (36.3)	0.527
No. of distal metastasis	1.5 ± 1.1	1.9 ± 1.2	1.5 ± 1.0	1.5 ± 1.1	0.064	2.0 ± 1.2	1.6 ± 1.1	1.6 ± 1.2	0.274	1.7 ± 1.4	1.5 ± 0.9	1.4 ± 1.0	0.292

Abbreviations: BMI, body mass index; COPD, chronic obstructive pulmonary disease; DM, diabetes mellitus; ECOG, eastern cooperative oncology group; EGFR, epidermal growth factor receptor.

## Data Availability

Data supporting reported results can be found at https://1drv.ms/u/s!AsRf0H3OWdB9gbIVN2v1yVO1US4kNw?e=iU6Zzf (accessed on 23 October 2021).
